# Impact of the Choice of Diagnostic Criteria and Growth Reference on the Prevalence of Extrauterine Growth Restriction in Extremely-Low-Birthweight Infants

**DOI:** 10.3390/children11080934

**Published:** 2024-07-31

**Authors:** Clara González López, Gonzalo Solís Sánchez, Belén Fernández Colomer, Laura Mantecón Fernández, Sonia Lareu Vidal, Sara Fernández Castiñeira, Ana Rubio Granda, Alicia Pérez Pérez, Marta Suárez Rodríguez

**Affiliations:** 1 Servicio de Neonatología, Área de Gestión Clínica de la Infancia y Adolescencia, Hospital Universitario Central de Asturias, 33011 Oviedo, Spain; 2 Instituto Investigación Sanitaria Principado de Asturias, ISPA, 33011 Oviedo, Spain; 3 Departamento de Medicina, Universidad de Oviedo, 33003 Oviedo, Spain; 4 Primary Care Interventions to Prevent Maternal and Child Chronic Diseases of Perinatal and Developmental Origin Network (RICORS), Instituto de Salud Carlos III, RD21/0012/0020, 28028 Madrid, Spain; 5 Área de Gestión Clínica de la Infancia y Adolescencia, Hospital Universitario Central de Asturias, 33011 Oviedo, Spain

**Keywords:** extrauterine growth restriction, extremely-low-birthweight infants, ELBW, Fenton, Olsen, INTERGROWTH-21st

## Abstract

Background and objectives: Variable diagnostic criteria and growth charts have been used for extrauterine growth restriction (EUGR). The objective was to assess the prevalence and concordance of EUGR in extremely-low-birthweight (ELBW) infants with the most frequent diagnostic criteria and growth charts. Materials and methods: An observational, retrospective and multicenter study was conducted from 2011 to 2020 including ELBW infants from the Spanish SEN1500 Network. EUGR prevalence was calculated at discharge using different definitions: cross-sectional (anthropometry less than the 10th centile), longitudinal (decrease of more than 1 SD from birth to discharge), “true” cross-sectional and “true” longitudinal (using the criteria previously described, excluding infants small for gestational age at birth). Concordance among Fenton, Olsen and INTERGROWTH-21st was assessed with Fleiss’ Kappa coefficient. Results: The prevalence of EUGR was variable with the different definitions and growth references studied in the 7914 ELBW infants included. Overall, it was higher with Fenton for all the EUGR criteria studied by weight and length. The agreement among growth charts was substantial (κ > 0.6) for all the definitions except for longitudinal EUGR by weight (moderate, κ = 0.578). Conclusions: The prevalence of EUGR was variable in our cohort with the different diagnostic criteria and growth charts. The agreement among charts was good for all the definitions of EUGR except longitudinal EUGR by weight.

## 1. Introduction

Postnatal growth of infants born preterm constitutes a field of controversy in neonatology [1]. Neither the optimal growth of the infant nor an ideal strategy for its evaluation have been described [2]. Serial measurements of weight, length and head circumference are the most frequently used in the clinical practice alongside their conversion to centiles and standard deviations compared to established growth charts [3]. The most common growth charts used in neonatology can be classified as reference charts, describing the fetal growth with the anthropometry at birth of the specific population where they were studied, such as Olsen [4] and Fenton [5], and standards of growth, describing the ideal postnatal growth of infants under optimal conditions, such as INTERGROWTH-21st [6]. Due to the peculiarities of the preterm population and their vulnerability to different comorbidities, it is particularly challenging to obtain “optimal” conditions for the establishment of standards of postnatal growth [7].

Extrauterine growth restriction is an extended and common term in both daily practice and the literature used to describe inadequate postnatal growth [8]. Different criteria have been proposed for EUGR, and variable timings for diagnosis have been proposed, from 36 weeks of corrected gestational age to discharge from hospital. EUGR has been described as anthropometry below the 10th percentile [9] (cross-sectional definition). An alternate longitudinal definition has been used to describe a loss of more than one [10] or two standard [11] deviations from birth. Recently, new criteria have been proposed (“true” cross-sectional and “true” longitudinal EUGR) for patients who meet the criteria for EUGR with the previously described definitions but were not small for gestational age at birth [12,13]. Traditionally, EUGR has been studied in relation to weight. However, several studies have found potential implications of EUGR by length [14,15] and head circumference [16,17,18].

Despite the frequency of use, variability in diagnostic criteria has been described, with discrepant criteria, times of diagnosis and growth references used in the literature [19], and the concept is not exempt from controversy [2]. One of the sources of controversy surrounding this term is the lack of consistency in the association of EUGR and significant outcomes. Variable results have been described for its association with poorer neurodevelopmental outcomes, with heterogeneity in the definitions for EUGR and the evaluation of neurodevelopmental outcomes complicating comparisons between the results obtained in the different studies [20].

The primary objective of this study is to describe the prevalence of extrauterine growth restriction with the most common definitions of the term (cross-sectional, longitudinal, “true” cross-sectional, and “true” longitudinal EUGR) for weight, length and head circumference in a multicenter cohort of extremely-low-birthweight (ELBW) infants in Spain. As a secondary objective, we propose the evaluation of concordance among some of the most frequently used growth charts (Fenton, Olsen and INTERGROWTH-21st) for the different definitions of EUGR studied.

## 2. Materials and Methods

This study was conducted with the ethics approval from “Comité de Ética de la Investigación del Principado de Asturias” with the reference number 2022.586. It was designed as an observational, retrospective and multicenter cohort study from the SEN1500 Network. This is a database from the Spanish Society of Neonatology that collects data from infants born very preterm or with a very low birthweight, delivered in level three neonatal intensive care units (NICUs) from Spain. For this study, the SEN1500 database was searched to include the subgroup of infants with a birthweight less than 1000 g (extremely-low-birthweight infants) born from 1 January 2011 to 31 December 2020. The data were collected in accordance with SEN1500 standardized operational definitions. All infants born ELBW and admitted at any of the participating NICUs, whose parents had provided written consent for inclusion in the database and the use of data for clinical studies, were eligible for inclusion. Exclusion criteria were set for major congenital anomalies at birth, congenital embryopathies causing growth impairment and gestational ages less than 24 weeks, as INTERGROWTH-21st is not available for those gestational ages.

The conversion of anthropometry to percentiles and z-scores for weight, length and head circumference for Fenton, Olsen and INTERGROWTH-21st was performed using the University of Calgary Fenton calculator application [21], Peditool Olsen bulk calculator [22] and INTERGROWTH-21st calculator application [23,24], respectively. For INTERGROWTH-21st calculations, two different references were used in the study, in accordance with the goals of the original project, using the reference of newborn size for preterm infants for anthropometry at birth and the chart of postnatal growth in preterm infants to evaluate subsequent postnatal growth. All of the above-mentioned resources are publicly available.

EUGR was defined in our study for weight, length and head circumference following the most frequently used criteria:

- Cross-sectional EUGR: anthropometry below the 10th centile for each growth reference at discharge from the hospital.
- Longitudinal EUGR: decrease of more than one standard deviation in anthropometry from birth to discharge from the NICU.
- “True” cross-sectional and “True” longitudinal EUGR: using the previously described criteria, respectively, including only infants who were not small for gestational age (SGA) at birth. Infants were considered SGA if the weight, length and head circumference were less than the 10th centile at birth.

All statistical analyses were performed using R (v 3.4.4, Open-Source International Collaborative), R Studio (v 1.1.463, Open-Source International Collaborative) and SPSS (V27). Basic descriptive statistics were used to characterize the data. As most of the quantitative variables were not normally distributed, median and interquartile ranges were used for quantitative variables and percentages were used for the qualitative variables. The primary outcome of interest (prevalence of EUGR) was evaluated with absolute frequencies and percentages. The secondary outcome of interest (concordance between Fenton, Olsen and INTERGROWTH-21st growth references) was assessed with Fleiss’ Kappa coefficient. The resulting coefficient was interpreted as very good agreement (κ > 0.8), substantial (0.6–0.8) and moderate (0.4–0.6).

## 3. Results

### 3.1. Study Population

**Figure 1:**
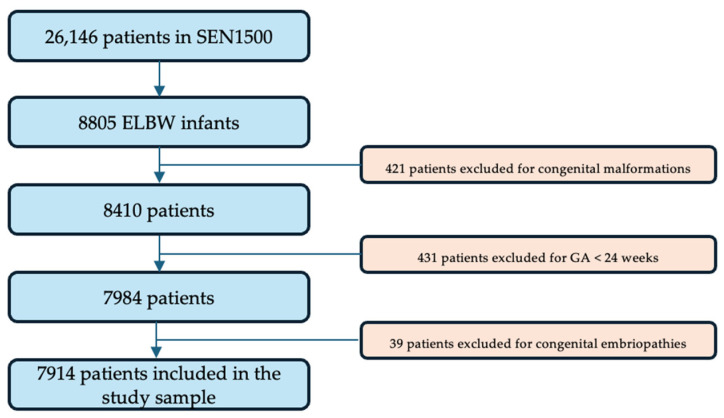
Flowchart of the population included in the study.

The reviewed database from the SEN1500 Network included 26,146 infants who were VLBW or born very preterm during the study period. From this sample 8805 infants were eligible for the study and 891 met the exclusion criteria previously specified: 421 patients had major congenital anomalies at birth, 39 patients had congenital embryopathies causing growth impairment such as cytomegalovirus infection and 431 patients were born at less than 24 weeks of gestational age. A total of 7914 ELBW infants were included in the final study sample after consideration of the inclusion and exclusion criteria, as presented in the flowchart in Figure 1.

### 3.2. Baseline Characteristics of the Study Sample

Considering maternal variables, infants were born to mothers with a median maternal age of 34 years (IQR 8). The pregnancy was non-spontaneous for 1466 patients (18.5%), and 877 gestations were multiple (11.1%). A total of 5586 (70.6%) mothers had received a complete course of prenatal steroids prior to delivery, 1572 (19.9%) received a partial course and 708 had no steroids prior to delivery.

From the study sample, 4046 patients were female (51.1%). A total of 5657 of the patients included were delivered by cesarean section. The median gestational age at delivery was 26 weeks (IQR 3). A total of 4148 patients required advanced resuscitation at delivery, consisting of intubation (4113 patients), chest compressions (698 patients) or, at least, one dose of epinephrine (450).

Among the most frequent comorbidities that the patients experienced during admission, we include the following: 5588 patients required invasive mechanical ventilation (70.6%), 5293 presented anemia requiring red blood cell transfusions (66.9%), 3816 patients were diagnosed with patent ductus arteriosus (48.3%), 3158 had shock requiring inotropes or vasoactives (39.9%), 946 (12%) developed necrotizing enterocolitis and 1159 (14.6%) presented intraventricular hemorrhage grades III or IV. The overall mortality in the study sample was 25.1%, with 1988 patients dying during the admission in the NICU. The overall survival without morbidities was 22.6% (1724 patients) from 7638 patients for whom this information was available.

### 3.3. Growth Pattern at Birth

The median birth weight was 800 g (IQR 200), the median length was 34 cm (IQR 3) and the median head circumference was 24 cm (IQR 2). At discharge, the median weight was 2225 g (IQR 1305), the median length was 44 cm (IQR 4) and the median head circumference was 33 cm (IQR 3). The median postmenstrual age at discharge (either discharge to home or time of death) was 38 weeks (IQR 7).

The prevalence of small for gestational age (SGA) was calculated using anthropometric measurements below the 10th percentile for each growth reference at birth, as described in Table 1.

### 3.4. Prevalence of EUGR with Different Diagnostic Criteria and Growth References

**Figure 2:**
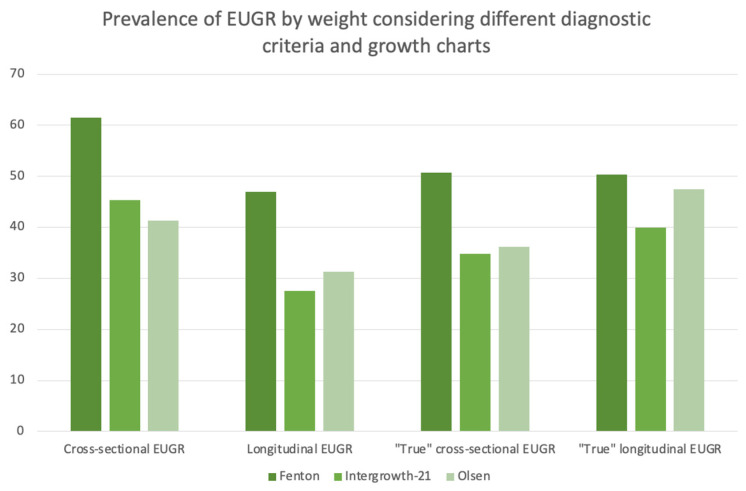
Prevalence of EUGR by weight with different diagnostic criteria and growth charts.

The impact of the choice of diagnostic criteria and growth reference on the prevalence of EUGR was assessed in the study sample by weight, length and head circumference, as presented in Table 2. As reflected in Table 2, the prevalence of EUGR drastically varied in our cohort and ranged from 13.1% using the cross-sectional EUGR definition based on the head circumference with Olsen (the lowest prevalence in our cohort) to 69.1% when considering the “true” longitudinal definition by length using Fenton (the highest prevalence of EUGR observed). Overall, the prevalence of EUGR was higher with Fenton for weight and length. In contrast, the prevalence of EUGR by head circumference was higher with INTERGROWTH-21st for most of the criteria studied. The variability of the prevalence of EUGR by weight according to the diagnostic criteria and growth charts is presented in Figure 2.

### 3.5. Concordance of SGA According to Different Growth References

The concordance of SGA according to different growth charts was evaluated using Fleiss’ Kappa coefficient and its 95% confidence interval, as presented in Table 3. There is very good agreement among the growth charts studied for the definition of SGA by weight, length and head circumference.

### 3.6. Concordance of EUGR According to Different Growth References

Fleiss’ Kappa coefficient and its 95% confidence interval were greater than 0.6 for all the definitions except for longitudinal EUGR by weight, as presented in Table 4. This corresponds to substantial agreement among growth charts for all the definitions except for longitudinal EUGR by weight, for which agreement was moderate.

## 4. Discussion

Despite the frequent use of the term extrauterine growth restriction both in the literature and clinical practice to describe infants whose post-birth growth does not meet expectations, there is significant variability in the diagnostic criteria used among different studies, as presented by Fenton et al. [5]. Due to the lack of consensus in the diagnostic criteria, variability in the prevalence of EUGR has previously been reported depending on the choice of criteria [11,25,26,27]. Moreover, it is important to highlight that, although several studies have evaluated EUGR in different populations (VLBW, extremely preterm infants, very preterm infants…), the literature specifically on the population included in our study, extremely-low-birthweight infants, remains scarce [28].

Evaluating EUGR by weight, variability was also found in the prevalence of EUGR with each definition and growth chart. Overall, the prevalence was higher with Fenton and lower with INTERGROWTH-21st for all the EUGR criteria studied by weight. This is consistent with the results reported by Kim et al. [28], who evaluated the prevalence of cross-sectional and longitudinal EUGR by weight in 2318 infants born before 28 weeks of gestational age from the Korean National Database and found a higher prevalence with Fenton compared to that with INTERGROWTH-21st. Similar results have been reported when evaluating very-low-birthweight infants, with a higher prevalence of EUGR using Fenton charts compared to INTERGROWTH-21st charts, as described by González-García et al. [26], Tuzun et at. [29], Reddy et al. [30] and El Rafei et al. [31]. Particularly, a study on prevalence and concordance for VLBW infants from the SEN1500 database, conducted using the same methodology as this study, reported that the prevalence of EUGR was higher in all the proposed definitions of EUGR using Fenton [32]. Moreover, this study described a lower prevalence of EUGR with all the criteria, growth charts and anthropometry (weight, length and head circumference) in VLBW compared to the latter study restricted to ELBW. On the contrary, Lan et al. [33] found comparable prevalences of cross-sectional EUGR by Fenton and INTERGROWTH-21st charts.

Considering EUGR by head circumference and length, we observed a lower prevalence of EUGR by head circumference with all the different criteria studied compared to EUGR defined by weight and length. This finding contrasts with the results presented by Kim et al. [28].

Concordance among different growth charts was at least substantial for all the definitions except for longitudinal EUGR by weight, for which the agreement was moderate. Several studies have evaluated the concordance of Fenton and INTERGROWTH-21st for the evaluation of EUGR, the majority conducted in different populations than that in our study. González-García et al. [26] evaluated VLBW infants and found good concordance between Fenton and INTERGROWTH-21st for all the criteria except for cross-sectional and “true” cross-sectional EUGR by weight and “true” longitudinal by length, for which agreement was moderate. Lan et al. [33] also described substantial concordance for the subgroup of infants born at less than 32 weeks of gestational age when comparing Fenton and INTERGROWTH-21st for longitudinal EUGR by weight, head circumference and length. In the study by González et al. [32] in VLBW from the Spanish national database, the concordance among Fenton, Olsen and INTERGROWTH-21st growth charts for EUGR by weight was only moderate for all the definitions except the cross-sectional definition. However, it was substantial or very good for the different criteria of EUGR by head circumference and length. In contrast, the same study found poor agreement between those two charts for cross-sectional EUGR in the same population. Peila et al. [11] have also reported low agreement between the cross-sectional and longitudinal definitions of EUGR.

It is important to highlight that EUGR is a concept that is not exempt from controversy. A group of experts has raised concerns about the potential that EUGR could lead to overdiagnosis [2] with no relation to significant clinical outcomes. Moreover, currently, there are limitations in comparing data on EUGR and neurodevelopment considering the significant variability in the criteria used in different studies [20]. Because of this, it is important to consider the choice of diagnostic criteria and growth chart and their impact on prevalence, as establishing the diagnosis of EUGR will likely have a direct impact in the clinical evaluation and follow-up of the patients.

The limitations of our study include the potential impact caused by the exclusion of infants born at less than 24 weeks of gestational age to allow for comparisons with INTERGROWTH-21st, as this reference does not include data for these gestational ages and the retrospective nature of the study with limited data on nutritional practices. Moreover, despite the differences in the prevalence of EUGR described in our study, no data were available on follow-up, and long-term outcomes of growth and neurodevelopment could not be evaluated. Further studies would be needed to assess the ideal definition of EUGR and the growth chart to predict these important outcomes and avoid potential overdiagnosis and studies with no clinical implications for the infant.

## 5. Conclusions

As presented in the data from our study, the choice of the definition of EUGR, growth reference and anthropometric measurements (weight, length or head circumference) had a drastic impact on the prevalence of EUGR in ELBW infants from our cohort. Despite differences in prevalence, the concordance among different growth charts was, at least, substantial for all the definitions except for longitudinal EUGR by weight, for which the agreement was moderate. Further studies need to be conducted to refine the diagnostic criteria of EUGR and an optimal growth chart, considering the variability depicted in this study.

## Figures and Tables

**Table 1:** Prevalence of small for gestational age (SGA) based on weight, length and head circumference with the different growth charts studied.

	Fenton	Olsen	INTERGROWTH-21st
Weight	2161 (27.3%)	2249 (28.4%)	2567 (32.4%)
Length	2074 (29.5%)	1658 (23.6%)	2421 (34.5%)
Head circumference	1889 (26.6%)	1472 (20.7%)	2048 (28.9%)

**Table 2:** Prevalence of EUGR by weight, length and head circumference with the different criteria proposed and growth charts.

	Fenton	Olsen	INTERGROWTH-21st
Weight			
Cross-sectional EUGR	4865 (61.5%)	3275 (41.3%)	3587 (45.3%)
Longitudinal EUGR	3717 (47%)	2477 (31.3%)	2175 (27.5%)
“True” cross-sectional EUGR	2862 of 5641 (50.7%)	1619 of 4967 (36.2%)	1548 of 4444 (34.8%)
“True” longitudinal	2839 of 5641 (50.3%)	2360 of 4967 (47.5%)	1771 of 4444 (39.9%)
Length			
Cross-sectional EUGR	4518 (57%)	3371 (42.5%)	4261 (53.8%)
Longitudinal EUGR	3665 (46.3%)	2912 (36.8%)	3276 (41.4%)
“True” cross-sectional EUGR	2625 of 4194 (62.6%)	2141 of 4004 (53.5%)	2231 of 3595 (62%)
“True” longitudinal EUGR	2896 of 4194 (69.1%)	2634 of 4004 (65.8%)	2164 of 3595 (60.2%)
Head circumference			
Cross-sectional EUGR	2476 (31.3%)	1113 (14.1%)	2739 (34,6%)
Longitudinal EUGR	1746 (22.1%)	1417 (17.9%)	1749 (22.1%)
“True” cross-sectional EUGR	1333 of 4371 (30.5%)	636 of 4180 (15.2%)	1473 of 3904 (37.7%)
“True” longitudinal EUGR	1588 of 4371 (36.3%)	1385 of 4179 (33.1%)	1404 of 3904 (36%)

**Table 3:** Fleiss’ Kappa coefficients and 95% confidence intervals for concordance amongst Fenton, Olsen and INTERGROWTH-21st growth charts for the definition of SGA.

	Fleiss’ Kappa	95% CI
SGA by weight	0.89	(0.87, 0.9)
SGA by length	0.82	(0.81, 0.83)
SGA by head circumference	0.83	(0.82, 0.85)

**Table 4:** Fleiss’ Kappa coefficients and 95% confidence intervals for each definition amongst Fenton, Olsen and INTERGROWTH-21st growth charts.

	Fleiss’ Kappa	95% CI
Weight		
Cross-sectional EUGR	0.710	(0.695, 0.725)
Longitudinal EUGR	0.578	(0.563, 0.593)
“True” cross-sectional EUGR	0.649	(0.631, 0.668)
“True” longitudinal EUGR	0.624	(0.605, 0.642)
Length		
Cross-sectional EUGR	0.843	(0.826, 0.859)
Longitudinal EUGR	0.695	(0.678, 0.711)
“True” cross-sectional EUGR	0.829	(0.809, 0.850)
“True” longitudinal EUGR	0.653	(0.633, 0.673)
Head circumference		
Cross-sectional EUGR	0.640	(0.624, 0.656)
Longitudinal EUGR	0.745	(0.728, 0.761)
“True” cross-sectional EUGR	0.631	(0.611, 0.651)
“True” longitudinal EUGR	0.751	(0.731, 0.771)

## Data Availability

All data, code and material will be available on request following publication.

## References

[1] Villar J., Giuliani F., Barros F., Roggero P., Coronado Zarco I.A., Rego M.A.S., Ochieng R., Gianni M.L., Rao S., Lambert A. (2018). Monitoring the Postnatal Growth of Preterm Infants: A Paradigm Change. Pediatrics.

[2] Fenton T.R., Cormack B., Goldberg D., Nasser R., Alshaikh B., Eliasziw M., Hay W.W., Hoyos A., Anderson D., Bloomfield F. (2020). “Extrauterine Growth Restriction” and “Postnatal Growth Failure” Are Misnomers for Preterm Infants. J. Perinatol..

[3] Tudehope D., Gibbons K., Cormack B., Bloomfield F. (2012). Growth Monitoring of Low Birthweight Infants: What References to Use?. J. Paediatr. Child Health.

[4] Olsen I.E., Groveman S.A., Lawson M.L., Clark R.H., Zemel B.S. (2010). New Intrauterine Growth Curves Based on United States Data. Pediatrics.

[5] Fenton T.R., Kim J.H. (2013). A Systematic Review and Meta-Analysis to Revise the Fenton Growth Chart for Preterm Infants. BMC Pediatr..

[6] Villar J., Cheikh Ismail L., Victora C.G., Ohuma E.O., Bertino E., Altman D.G., Lambert A., Papageorghiou A.T., Carvalho M., Jaffer Y.A. (2014). International Standards for Newborn Weight, Length, and Head Circumference by Gestational Age and Sex: The Newborn Cross-Sectional Study of the INTERGROWTH-21st Project. Lancet.

[7] Cormack B.E., Embleton N.D., van Goudoever J.B., Hay W.W., Bloomfield F.H. (2016). Comparing Apples with Apples: It Is Time for Standardized Reporting of Neonatal Nutrition and Growth Studies. Pediatr. Res..

[8] Gounaris A.K., Sokou R., Gounari E.A., Panagiotounakou P., Grivea I.N. (2023). Extrauterine Growth Restriction and Optimal Growth of Very Preterm Neonates: State of the Art. Nutrients.

[9] Clark R.H., Thomas P., Peabody J. (2003). Extrauterine Growth Restriction Remains a Serious Problem in Prematurely Born Neonates. Pediatrics.

[10] Shah P.S., Wong K.Y., Merko S., Bishara R., Dunn M., Asztalos E., Darling P.B. (2006). Postnatal Growth Failure in Preterm Infants: Ascertainment and Relation to Long-Term Outcome. J. Perinat. Med..

[11] Peila C., Spada E., Giuliani F., Maiocco G., Raia M., Cresi F., Bertino E., Coscia A. (2020). Extrauterine Growth Restriction: Definitions and Predictability of Outcomes in a Cohort of Very Low Birth Weight Infants or Preterm Neonates. Nutrients.

[12] Figueras-Aloy J., Palet-Trujols C., Matas-Barceló I., Botet-Mussons F., Carbonell-Estrany X. (2020). Extrauterine Growth Restriction in Very Preterm Infant: Etiology, Diagnosis, and 2-Year Follow-Up. Eur. J. Pediatr..

[13] Zhao T., Feng H.-M., Caicike B., Zhu Y.-P. (2021). Investigation Into the Current Situation and Analysis of the Factors Influencing Extrauterine Growth Retardation in Preterm Infants. Front. Pediatr..

[14] Meyers J.M., Tan S., Bell E.F., Duncan A.F., Guillet R., Stoll B.J., D’Angio C.T. (2019). Eunice Kennedy Shriver National Institute of Child Health and Human Development Neonatal Research Network Neurodevelopmental Outcomes among Extremely Premature Infants with Linear Growth Restriction. J. Perinatol..

[15] Sammallahti S., Pyhälä R., Lahti M., Lahti J., Pesonen A.-K., Heinonen K., Hovi P., Eriksson J.G., Strang-Karlsson S., Andersson S. (2014). Infant Growth after Preterm Birth and Neurocognitive Abilities in Young Adulthood. J. Pediatr..

[16] Ehrenkranz R.A., Dusick A.M., Vohr B.R., Wright L.L., Wrage L.A., Poole W.K. (2006). Growth in the Neonatal Intensive Care Unit Influences Neurodevelopmental and Growth Outcomes of Extremely Low Birth Weight Infants. Pediatrics.

[17] Belfort M.B., Rifas-Shiman S.L., Sullivan T., Collins C.T., McPhee A.J., Ryan P., Kleinman K.P., Gillman M.W., Gibson R.A., Makrides M. (2011). Infant Growth before and after Term: Effects on Neurodevelopment in Preterm Infants. Pediatrics.

[18] Franz A.R., Pohlandt F., Bode H., Mihatsch W.A., Sander S., Kron M., Steinmacher J. (2009). Intrauterine, Early Neonatal, and Postdischarge Growth and Neurodevelopmental Outcome at 5.4 Years in Extremely Preterm Infants after Intensive Neonatal Nutritional Support. Pediatrics.

[19] Fenton T.R., Chan H.T., Madhu A., Griffin I.J., Hoyos A., Ziegler E.E., Groh-Wargo S., Carlson S.J., Senterre T., Anderson D. (2017). Preterm Infant Growth Velocity Calculations: A Systematic Review. Pediatrics.

[20] González-López C., Solís-Sánchez G., Lareu-Vidal S., Mantecón-Fernández L., Ibáñez-Fernández A., Rubio-Granda A., Suárez-Rodríguez M. (2024). Variability in Definitions and Criteria of Extrauterine Growth Restriction and Its Association with Neurodevelopmental Outcomes in Preterm Infants: A Narrative Review. Nutrients.

[21] Calculators & Apps | Welcome to the Fenton Preterm Growth Chart Site | University of Calgary. https://ucalgary.ca/resource/preterm-growth-chart/calculators-apps.

[22] PediTools Universal Calculator. https://peditools.org/peditools_universal/.

[23] Newborn Size for Very Preterm Infants • INTERGROWTH-21st. https://intergrowth21.tghn.org/very-preterm-size-birth/.

[24] INTERGROWTH-21st http://intergrowth21.ndog.ox.ac.uk/preterm.

[25] Shan H.M., Cai W., Cao Y., Fang B.H., Feng Y. (2009). Extrauterine Growth Retardation in Premature Infants in Shanghai: A Multicenter Retrospective Review. Eur. J. Pediatr..

[26] González-García L., García-López E., Fernández-Colomer B., Mantecón-Fernández L., Lareu-Vidal S., Suárez-Rodríguez M., Arias-Llorente R.P., Solís-Sánchez G. (2021). Extrauterine Growth Restriction in Very Low Birth Weight Infants: Concordance Between Fenton 2013 and INTERGROWTH-21st Growth Charts. Front. Pediatr..

[27] Avila-Alvarez A., Solar Boga A., Bermúdez-Hormigo C., Fuentes Carballal J. (2018). Extrauterine growth restriction among neonates with a birthweight less than 1500 grams. An. De Pediatría (Engl. Ed.).

[28] Kim Y.-J., Shin S.H., Cho H., Shin S.H., Kim S.H., Song I.G., Kim E.-K., Kim H.-S. (2021). Extrauterine Growth Restriction in Extremely Preterm Infants Based on the Intergrowth-21st Project Preterm Postnatal Follow-up Study Growth Charts and the Fenton Growth Charts. Eur. J. Pediatr..

[29] Tuzun F., Yucesoy E., Baysal B., Kumral A., Duman N., Ozkan H. (2018). Comparison of INTERGROWTH-21 and Fenton Growth Standards to Assess Size at Birth and Extrauterine Growth in Very Preterm Infants. J. Matern. Fetal Neonatal Med..

[30] Reddy K.V., Sharma D., Vardhelli V., Bashir T., Deshbotla S.K., Murki S. (2021). Comparison of Fenton 2013 Growth Curves and Intergrowth-21 Growth Standards to Assess the Incidence of Intrauterine Growth Restriction and Extrauterine Growth Restriction in Preterm Neonates ≤32 Weeks. J. Matern. Fetal Neonatal Med..

[31] El Rafei R., Jarreau P.-H., Norman M., Maier R.F., Barros H., Reempts P.V., Pedersen P., Cuttini M., Zeitlin J., EPICE Research Group (2021). Variation in Very Preterm Extrauterine Growth in a European Multicountry Cohort. Arch. Dis. Child. Fetal Neonatal Ed..

[32] Gonzalez Lopez C., Solís Sánchez G., Fernández Colomer B., Mantecón Fernández L., Lareu Vidal S., Arias Llorente R.P., Ibáñez Fernández A., González García L.G., Suárez Rodríguez M. (2024). Extrauterine Growth Restriction in Very-Low-Birthweight Infants: Prevalence and Concordance According to Fenton, Olsen, and INTERGROWTH-21st Growth Charts in a Multicenter Spanish Cohort. Eur. J. Pediatr..

[33] Lan S., Fu H., Zhang C., Chen Y., Pan L., Song S., Wang Y., Hong L. (2023). Comparison of Intergrowth-21st and Fenton Growth Standards to Evaluate and Predict the Postnatal Growth in Eastern Chinese Preterm Infants. Front. Pediatr..

